# A methodological evaluation of the determination of critical oxygen threshold in an estuarine teleost

**DOI:** 10.1242/bio.045310

**Published:** 2019-11-07

**Authors:** Benjamin Negrete, Andrew J. Esbaugh

**Affiliations:** Department of Marine Science, Marine Science Institute, The University of Texas at Austin, Port Aransas, TX 78373, USA

**Keywords:** Hypoxia, Tolerance, Fish, Respirometry, Nitrogen, P_crit_

## Abstract

One measure of hypoxia tolerance is the critical oxygen threshold, P_crit_, which is the point where standard metabolism can no longer be maintained through aerobic processes. Traditionally, P_crit_ was determined using closed respirometry, whereby the fish's respiration naturally lowered O_2_. More recently, intermittent flow techniques have been adopted, where N_2_ is used to displace O_2_, which ostensibly reduces end-product build-up. This study used a paired design on the marine teleost, red drum. P_crit_ is comparable between closed (4.6±0.2 kPa; mean±s.e.m.) and intermittent flow (4.4±0.2 kPa; mean±s.e.m.) respirometry. pCO_2_, ammonia and pH changes within the chamber were measured prior to the onset of P_crit_ and at the end of a typical P_crit_ trial and revealed changes in water chemistry in both closed and intermittent flow. P_crit_ values were similar in both methods of hypoxia induction regardless of subsequent water chemistry changes that occurred in both methods.

## Abstract (precis)

**Summary:** The two leading methods of measuring the critical oxygen threshold in fishes are similar in their estimations, regardless of changes to water chemistry.

## INTRODUCTION

Hypoxia is a common environmental stress for aquatic organisms, and researchers’ ability to quantify inter- and intraspecies hypoxia tolerance is important when making predictions about species resilience. Two common metrics of whole-animal hypoxia tolerance are the time to loss of equilibrium (LOE), and the critical oxygen threshold (P_crit_). The former refers to the amount of time an animal can survive when forced to rely on anaerobic metabolism, while P_crit_ refers to the ambient oxygen level whereby an animal can no longer maintain energetic costs on aerobic metabolism alone. P_crit_ is considered a powerful tool to assess hypoxia tolerance, with a lower P_crit_ considered more hypoxia tolerant (e.g. Mandic et al., 2008). It is well-known that hypoxia is a limiting cardiorespiratory stress and constrains maximum metabolic rate (MMR): as dissolved oxygen (DO) declines, MMR will also decline until it intersects with the standard metabolic rate (SMR). SMR is the baseline amount of energy required to sustain vital function (Clark et al., 2013; Chabot et al., 2016). This intersection represents P_crit_, and is defined as the point where O_2_ supply is insufficient to maintain SMR. When DO is below P_crit_, animals increase reliance on unsustainable anaerobic pathways for adenosine triphosphate (ATP) production (Mandic et al., 2013; Claireaux and Chabot, 2016; Chabot et al., 2016; Rogers et al., 2016). The merits of P_crit_ were recently debated in the scientific literature. As argued by Regan et al. (2019), the value of P_crit_ as a measure of hypoxia tolerance is rooted in the Fry concept of aerobic metabolic scope theory (reviewed by Claireaux and Chabot, 2016). Yet the methodological concerns argued by Wood (2018) remain true, and represent a particularly troubling aspect of interspecies and inter-study comparisons.

The methodological inconsistencies with P_crit_ estimation are threefold: (1) the method of reducing O_2_ within the respirometry system (closed versus intermittent flow), (2) the metabolic rate measurements used to anchor P_crit_ (i.e. standard versus routine metabolic rate, RMR), and (3) the mathematical approaches used to calculate P_crit_. With regard to the former, the classical method uses closed circuit respirometry, whereby the chamber is sealed from incurrent and excurrent flow and fish respiration depletes O_2_. This method is simple, but it has been argued that build-up of CO_2_ and ammonia are confounding factors that influence P_crit_ (e.g. Snyder et al., 2016). An alternate intermittent flow method uses nitrogen gas to remove O_2_ from the solution prior to entering the respirometry chamber (Rogers et al., 2016; Snyder et al., 2016). Flow into and out of the chamber is maintained, which eliminates the build-up of CO_2_ and ammonia. Intermittent flow has the additional benefit of controlling time spent at various DO saturations, and the rate of O_2_ depletion. The latter can enhance a fish's ability to acclimate to hypoxia and result in a lower P_crit_ (Regan and Richards, 2017). This has been argued to provide more precise estimates of P_crit_ (Snyder et al., 2016), although at the cost of additional equipment and a more complex experimental setup (Svendsen et al., 2016).

While there remains debate in the scientific community regarding the best practices for reaching P_crit_, there is a growing perception that intermittent flow protocols are preferable (e.g. Snyder et al., 2016; Svendsen et al., 2016; Herbert et al., 2017; Wong et al., 2018). This perception has likely been buoyed, in part, by the fact that intermittent flow is undoubtedly the best method to determine SMR (Steffensen, 1989; Chabot et al., 2016). Yet the experimental data in support of intermittent flow P_crit_ methods are less equivocal. Snyder et al. (2016) found that intermittent flow resulted in lower P_crit_ using an unpaired design and no measure of waste build-up. Regan and Richards (2017) demonstrated no difference between methodologies when anchoring to RMR – a measurement which includes ambient swimming activity – in the freshwater goldfish (*Carassius auratus auratus*, Linnaeus 1758) despite changes of water chemistry in closed respirometry. Additionally, a thorough meta-analysis of P_crit_ methodologies could not identify any difference between methods in studies that used both on the same species (Rogers et al. 2016). However, the authors acknowledged the need for further experimentation owing to a limited number of available studies that employ both techniques on the same species. Further, Rogers et al. (2016) modeled the predicted changes in CO_2_ depending on the starting pH and salinity in the chamber to illustrate the importance of starting water chemistry and its changes inside the chamber. A recent paper by Reemeyer and Rees (2019) targeted various mathematical methods to calculate the P_crit_ and found a significant difference between methods. Thus, the aim of this study was to address the methodological concerns regarding P_crit_ in closed versus intermittent flow methods, with specific attention on the changes in water chemistry (pH, pCO_2_ and total ammonia) for both treatments prior to the onset P_crit_, and at the end of a P_crit_ trial. Furthermore, we address this question in the context of varying methods of defining baseline metabolic rates and mathematical calculations of P_crit_. The estuarine teleost red drum (*Sciaenops ocellatus*, Linnaeus 1766) was chosen as a study species because their distribution is known to overlap with the extensive oxygen minimum zones found in the northern Gulf of Mexico, and have previously shown to be amenable to respirometry techniques (Ern et al., 2016; Pan et al., 2016; Pan et al., 2017; Ern and Esbaugh, 2018).

## RESULTS

**Fig. 1.**
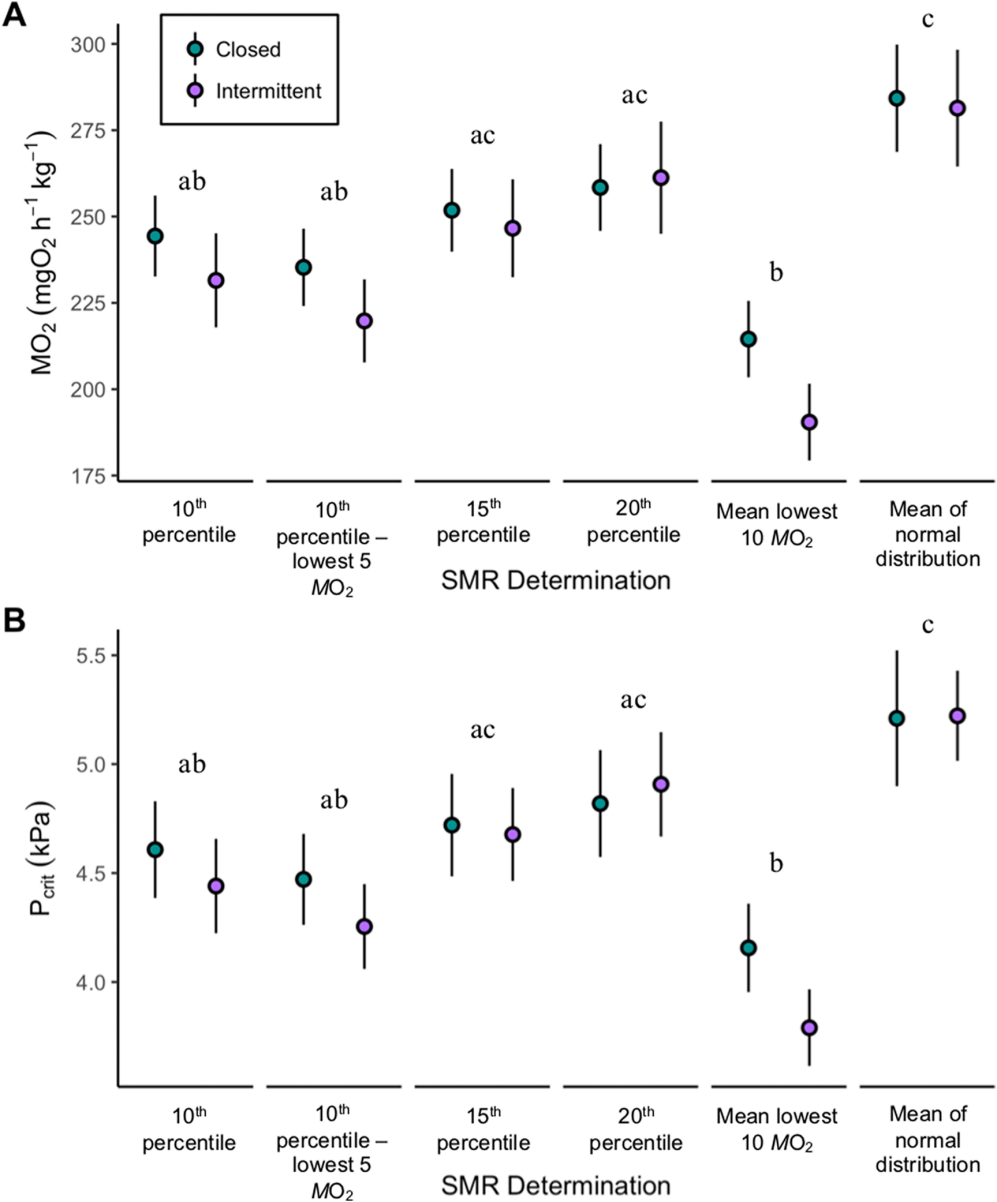
**Comparison of SMR and P_crit_ estimates obtained** **from closed circuit (*N*=11) and intermittent flow (*N*=9) protocols using different mathematical approaches.** (A) Average final SMR measurements for all fish. SMR was determined by intermittent flow and groups here indicate fish assigned to P_crit_ treatment groups. (B) Final P_crit_ measurements determined by closed and intermittent flow and how it varies across SMR determinations. Data are mean±s.e.m. Different letters denote statistically significant groups based on mathematical approach (two-way repeated measures ANOVA; *P*≤0.05). No differences were detected between closed circuit and intermittent flow methods.

There was no significant difference in estimates of SMR between initial or final (*P*=0.73, *F*=0.12, two-way ANOVA), or between intermittent flow and closed circuit trials (Fig. 1A, *P*=0.12, *F*=2.47; two-way ANOVA). A two-way repeated measures (RM) ANOVA revealed that SMR varied significantly with mathematical method of estimation (Fig. 1A, *P<*0.01, *F*=88.928), but not between respirometry methods (*P*=0.935, *F*=0.007).

P_crit_ was calculated relative to SMR determined by the lowest 10th percentile. The mean initial trial P_crit_ for all fish was 5.0±0.2 kPa (mean±s.e.m.; *N*=20). The average final P_crit_ for closed circuits was 4.6±0.2 kPa (mean±s.e.m.) versus intermittent flow of 4.4±0.2 kPa (pooled 4.5±0.2 kPa, mean±s.e.m.). P_crit_ showed a significant decrease between the initial and final measurement (*P*<0.01, *F*=12.28; two-way ANOVA). Closed circuit trials lasted an average of 73.8±7 min (mean±s.e.m.; initial, *N*=20) and 61±4.5 min (mean±s.e.m.; final, *N*=11). Intermittent flow trials lasted an average of 233±10.2 min (mean±s.e.m.; final, *N*=9). Fish exhibited LOE in both types of respirometry with equal number of occurrences (data not shown). A second series of analysis demonstrated that the similarity in final P_crit_ measurements between closed circuit and intermittent flow was consistent regardless of mathematical methods for SMR (Fig. 1B; *P*=0.81; *F*=0.058; two-way RM ANOVA; Student's *t*-test results in Table 1). However, there was a difference in the pooled P_crit_ values when using different methods of SMR calculations (Fig. 1B; *P<*0.01, *F*=88.928; two-way RM ANOVA) and an interaction between SMR determination and treatment (*P*<0.01, *F*=4.123; two-way RM ANOVA).

**Table 1.**
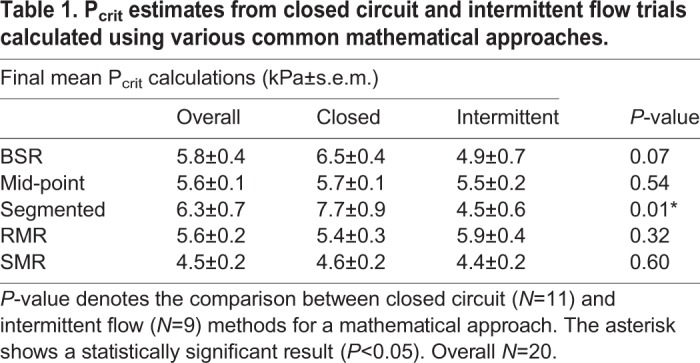
**P_crit_ estimates from closed circuit and intermittent flow trials calculated using various common mathematical approaches.**

**Fig. 2.**
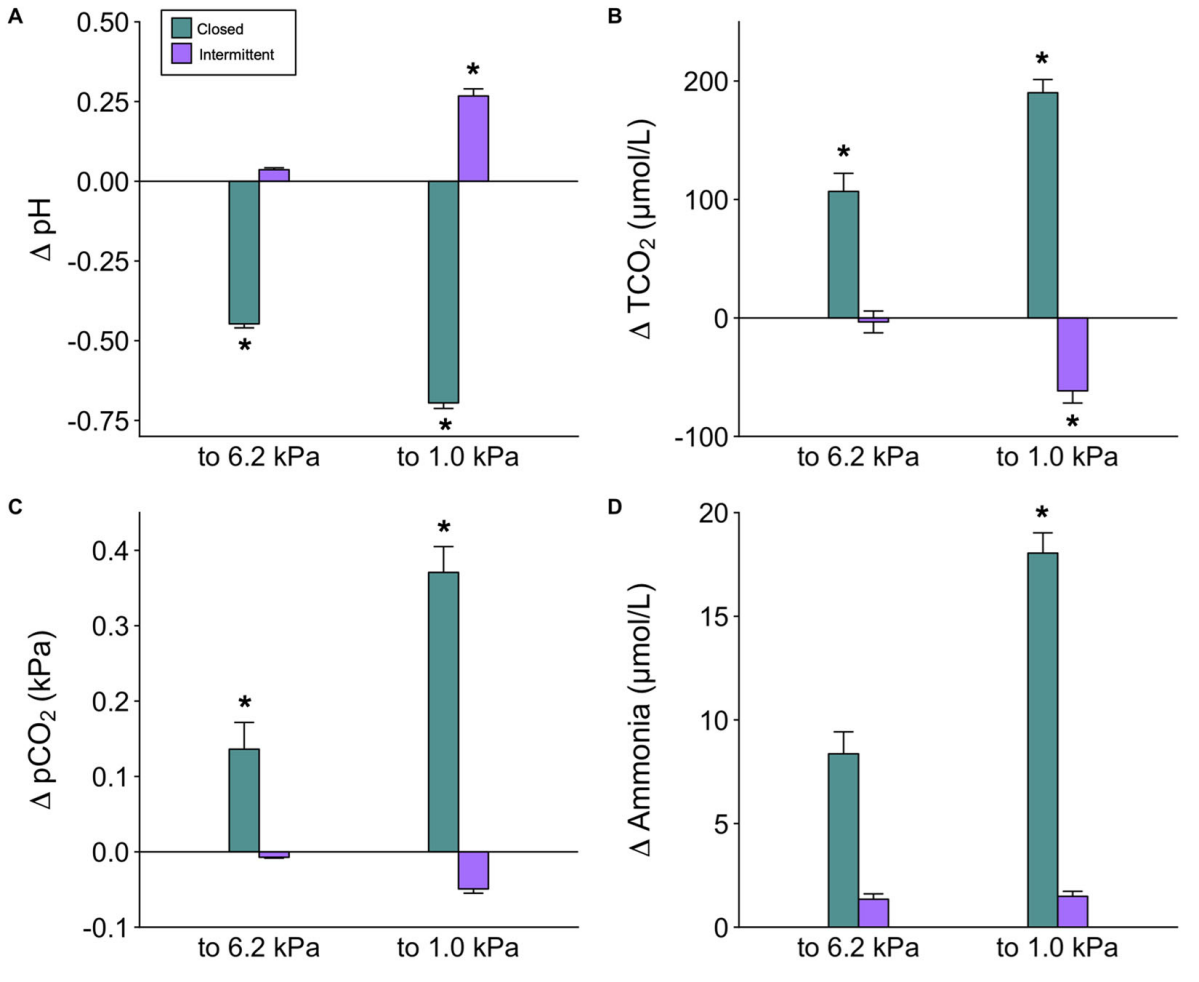
**Measurements of waste accumulation inside a respirometry** **chamber during closed (*N*=16) and intermittent flow (*N*=16) P_crit_ trials.** Differences are calculated from starting water chemistry of a P_crit_ trial (the end of an SMR trial) for (A) pH, (B) total CO_2_, (C) partial pressure of CO_2_ and (D) total ammonia. Data are mean±s.e.m. (two-way ANOVA, *P<0.01).

**Fig. 3.**
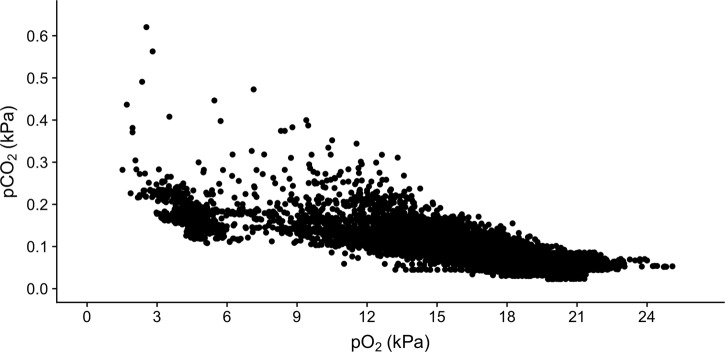
**Representative plot demonstrating the relationship between dissolved oxygen and CO_2_ in the coastal regions of the northern Gulf of Mexico.** Data are from Cat Point, FL, USA, from the Apalachicola Bay NERR. Each point represents water quality measurements taken every 15 min during the summer of 2012.

The quantification of waste build-up for the two protocols at various time points is presented in Table 2. For all four variables there was a significant effect of time point, treatment, as well as a significant interaction between the two factors (*P*<0.01; two-way ANOVA). As expected, the closed circuit method resulted in significant increases in ammonia and pCO_2_, with a concurrent decrease in pH (Fig. 2). Interestingly, a higher proportion of the waste build-up occurred after passing P_crit_ as evident by the differences at 6.2 kPa and 1.0 kPa. Changes in water chemistry were also observed during intermittent flow protocols (Fig. 2). By the end of the trial (1.0 kPa) the chamber pH had increased significantly (*P=*0.03; two-way ANOVA) likely owing to displacement of CO_2_ by nitrogen. Note that the observed decrease in pCO_2_ at 1.0 kPa pO_2_ was just outside of statistical significance (*P*=0.06; two-way ANOVA). No significant changes were noted at the sampling point just prior to P_crit_, and no significant increases were observed in ammonia at any sampling point. For purposes of comparison, the relationship between pCO_2_ and DO from a National Estuarine Research Reserve (NERR) station in the northern Gulf of Mexico – a station that regularly exhibits severe hypoxia – is provided in Fig. 3. In this station, when DO was between 4 and 7 kPa (i.e. the approximate range of observed P_crit_) the median pCO_2_ was 0.17 kPa (1679 µatm).

**Table 2.**
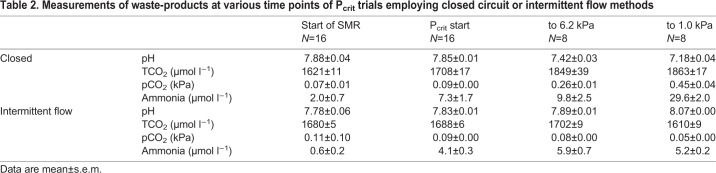
**Measurements of waste-products at various time points of P_crit_ trials employing closed circuit or intermittent flow methods**

## DISCUSSION

There is a growing perception that the intermittent flow method to estimate P_crit_ is superior to the long used closed circuit method because it eliminates the build-up of toxic metabolic end products within the respirometry chamber, which are hypothesized to cause artificially high P_crit_ estimates. Here, we used a paired experimental design to demonstrate that P_crit_ estimates did not differ between methods. In fact, P_crit_ estimates were almost identical between methodologies when calculated relative to SMR. These data provide important experimental support for the conclusions made by the meta-analysis of Rogers et al. (2016), which the authors acknowledge were drawn from a relatively small dataset. Additionally, the various methods of calculating SMR and P_crit_ showed similarities between methods confirming the findings of Reemeyer and Rees (2019) on the Gulf killifish *Fundulus grandis*, Baird & Girard 1853.

As mentioned above, the primary concern related to closed circuit methodologies relates to waste build-up; however, the extent of waste build-up is rarely measured nor placed in the context of impaired oxygen delivery. At this point, it is important to remember that best practices for determining SMR – the prerequisites for estimating P_crit_ – state that an animal should be fasted for 24–48 h prior to measurement to remove any influence of specific dynamic action (Chabot et al., 2016). As such, it is not surprising that total ammonia was found to be low during closed circuit trials and unlikely to interfere with P_crit_ determination. For example, water samples taken from the closed chamber just prior to reaching P_crit_ were only 9.8 µmol l^−1^ versus 5.9 µmol l^−1^ in the intermittent flow system (Table 2). A more marked difference in total ammonia was observed at the end of the P_crit_ trial; however, most of this occurred after P_crit_ had been passed (Table 2, Fig. 2D). The increase in apparent ammonia excretion between 6.2 kPa and 1.0 kPa of the closed trial (Table 2, Fig. 2D) is curious; however, it seems likely that it relates to the increased H^+^ excretion rates that will accompany anaerobic metabolism. The positive relationship between H^+^ excretion and ammonia excretion is well documented and relates to the metabolon-style interactions of Na^+^H^+^ exchanger 3 (NHE3) and the Rh ammonia transporters (Wright et al., 2016; Chen et al., 2017). Overall, our findings relating to ammonia build-up are in line with recent work by Regan and Richards (2017), which reported final total ammonia concentrations of ∼47 µmol l^−1^ in closed circuit trials. Given that the average acute ammonia toxicity for marine teleosts is 109.2 µmol l^−1^ (converted from mg l^−1^, reviewed in Randall and Tsui, 2002; Eddy, 2005), it seems unlikely that short-term exposure to these ammonia levels would impact oxygen supply.

In contrast to ammonia, there was a more pronounced increase in pCO_2_ during a closed circuit trial. The average pCO_2_ reached 0.26 kPa just prior to P_crit_ (6.2 kPa pO_2_) and 0.45 kPa at the end of a typical trial (1.0 kPa pO_2_), which represented an increase of approximately 0.17 kPa and 0.37 kPa, respectively (Fig. 2C). These increases were accompanied by a drop in pH to 7.42 and 7.18, respectively (Fig. 2A). Our pCO_2_ measurements agree with data reported by Regan and Richards (2017), and Rogers et al. (2016), the latter of which proposed a model highlighting potential changes in water chemistry in a closed trial with particular consideration to starting water pCO_2_ and salinity. Interestingly, these values are in range of recent work on ocean acidification, which can also provide useful context when attempting to interpret impacts on P_crit_. The available evidence would suggest that this pCO_2_ may be sufficient to generate a small respiratory acidosis (Esbaugh et al. 2012; Esbaugh et al., 2016; Ern and Esbaugh, 2018), yet to impact P_crit_ it would need to overwhelm the β-adrenergic sodium-proton exchanger (NHE) in the red blood cell (reviewed by Esbaugh, 2018). It seems unlikely that this pCO_2_ build-up would overwhelm β-NHE and impair hemoglobin (Hb)–O_2_ binding affinity in red drum. Similarly, it is unlikely that SMR would be impacted by these pCO_2_ levels (reviewed by Lefevre, 2016; Esbaugh, 2018). This is also supported by Regan and Richards (2017), who observed a final pCO_2_ of ∼0.8 kPa with no effects on P_crit_. It is also noteworthy that intermittent flow is not immune to mild respiratory disturbances. Our data demonstrate that N_2_ also displaces CO_2_ and lowers pCO_2_ by ∼0.01 kPa prior to P_crit_, and by half at the conclusion of the trial (Table 2). There is less information available on the consequences of a mild respiratory alkalosis; however, this level of change also seems unlikely to affect Hb–O_2_ binding affinity. As pointed out by Wood (2018), environmental hypoxia is commonly associated with elevated pCO_2_ since hypoxia is generated through metabolic processes of other organisms in an environment. In fact, the levels of pCO_2_ produced during a closed circuit trial may be representative to those common in the marine environment (Fig. 3). This is an important yet often overlooked aspect of the methodological debates surrounding P_crit_. If the purpose of study is to place hypoxia tolerance in an environmental context, the elevated pCO_2_ during closed circuit trials should be viewed as a benefit. While it is important to note that intermittent flow still has an important place when disentangling mechanisms driving P_crit_ (e.g. β-NHE activity), closed circuit may provide a more representative estimate for ecological applications.

Prior work has suggested that the rate of hypoxia induction may also impact P_crit_ estimation owing to the time required for physiological responses to occur (Regan and Richards, 2017). These investigations demonstrated that longer trials (480 min; six times longer) coincided with lower P_crit_ estimates in goldfish, which was due in part to the shedding of interlamellar cell mass. Snyder et al. (2016) found a decrease in P_crit_ in intermittent flow, and it is possible that this was due to the longer trial duration. Our data in red drum tested over 61 min±4.5 (mean±s.e.m.) showed similar P_crit_ as individuals tested over 233 min±10 (mean±s.e.m.). While our protocols only differed by a fourfold duration, we believe the differences lie in the species' biology. Unlike goldfish, red drum do not exhibit interlamellar cell mass. In fact, a 3-week hypoxia acclimation (30% O_2_ DO, 6.2 kPa) did not result in significant changes in gill morphology in red drum (Pan et al., 2017). Regardless, it seems prudent to consider the study organism’s biology – especially that pertaining to the oxygen supply cascade – when planning P_crit_ studies. Similarly, researchers should optimize organism and chamber sizes for longer protocols, while remaining within the best practices for respirometry experiments (e.g. a larger chamber-to-body-mass ratio; Svendsen et al., 2016).

**Fig. 4.**
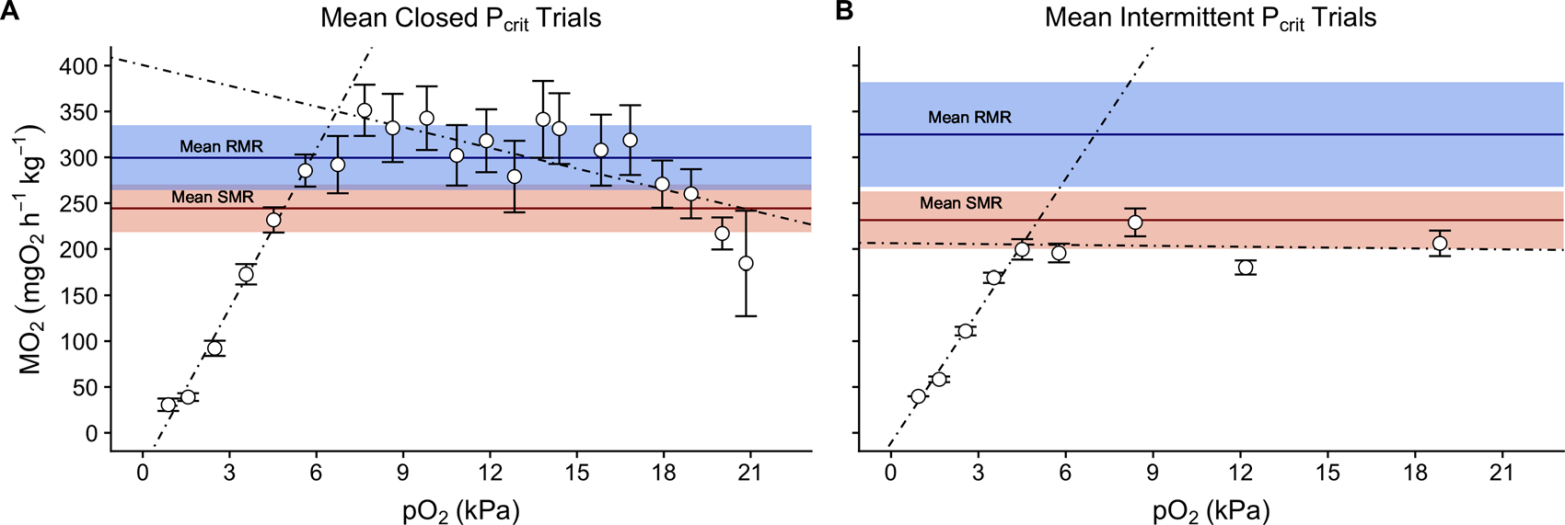
**Plots of the mean *M*O_2_ versus pO_2_ for final measurements of P_crit_.** P_crit_ traces for closed circuit (*N*=11, A) and intermittent flow (*N*=9, B) are averaged within their respective trials and presented as mean±s.e.m. (white dots). Mean RMR (blue) and SMR (red) were determined from 24 h of intermittent flow data collected immediately before the P_crit_ trial, with the respective shaded area representing the 95% confidence intervals. The dotted lines represent the respective linear regressions, as calculated from R packages rMR and respR, with the intersection between the two denoting the mean broken line estimate for P_crit_.

A final set of methodological consideration for estimating P_crit_ is the mathematical approaches to data analysis. While the importance of this should be obvious, it is highlighted by the fact that the overall conclusions of this study can vary depending on the applied calculations. Both the BSR and segmented line approach resulted in a significantly lower final P_crit_ using the intermittent flow protocol (Table 1). This is purely the product of differences in the *M*O_2_ measures at higher pO_2_ (>6.2 kPa) of the P_crit_ trace, which is depicted in Fig. 4. In fact, the variance in *M*O_2_ of P_crit_ traces was pointed out in Wood's criticism of P_crit_ (Wood, 2018). The BSR and segmented line approaches assume that the response of *M*O_2_ to declining pO_2_ consists of two linear relationships, with P_crit_ as the transition between them, disregarding the 24 h of *M*O_2_ collected prior to P_crit_ trials. These relationships are not necessarily met by real data and said to not be applicable to 25% of datasets (Rogers et al., 2016; Wood, 2018). At this point it is also crucial to revisit the definition of P_crit_ as outlined in the Fry paradigm of aerobic metabolism (reviewed by Claireaux and Chabot, 2016), which highlights that P_crit_ is the point at which SMR can no longer be maintained by aerobic pathways alone. This was also pointed out recently by Regan et al. (2019). Given these facts, it seems that the practice of anchoring P_crit_ to SMR is preferred, despite its scarcity in the literature (Rogers et al., 2016). Reemeyer and Rees (2019) showed that P_crit_ calculation can vary with calculation methods, and recommended the use of SMR for comparable and repeatable measures of P_crit_. Regan and Richards (2017) estimated P_crit_ against RMR calculated during early normoxic portions of the P_crit_ trial, rather than SMR as performed here. While no changes in RMR were noted between the various protocols, it is unclear if the effects of duration would be as dramatic when compared to SMR. In a best-case scenario, the use of RMR would provide an estimate of P_crit_ similar to that of SMR (e.g. Fig. 4B), but a worst-case scenario would result in a vast overestimate (Fig. 4A). It seems reasonable to simply opt to apply the SMR approach when at all possible. Importantly, there are also many available calculations that are employed for SMR (Fig. 1). But our analysis suggests that the estimates of hypoxia tolerance as measured by P_crit_ in intermittent flow versus closed circuit trials were similar regardless of the SMR calculation (Fig. 1, Table 1). While this provides a degree of certainty in the estimate for comparative purposes within a study, we would recommend that researchers report a suite of SMR calculations to aid cross study comparisons.

Overall, the experiments described here suggest that closed circuit and intermittent flow methods result in comparable P_crit_ estimates in red drum, and that the end-product build-up during closed circuit trials is not sufficient to impair the oxygen supply cascade. In fact, closed circuit protocols have the benefit of more accurately representing a natural hypoxic environment with respect to pCO_2_, while also being less mechanically complex and more cost effective. This is not a condemnation of intermittent flow, as there are several instances where it can benefit a research question (e.g. ion poor waters). We also stress that it is important to consider both the ambient water chemistry and the organism's biology when planning P_crit_ experiments, particularly as the latter pertains to dynamic morphological changes in response to hypoxia. Finally, we would stress that an accurate measure of SMR is the most crucial, and often overlooked, factor when determining P_crit_.

## MATERIALS AND METHODS

### Fish

Red drum were purchased from Ekstrom Aquaculture LLC (Palacios, USA) in April 2018 and held at the Fisheries and Mariculture Laboratory at The University of Texas at Austin Marine Science Institute (Port Aransas, USA). Following a 1-month acclimation period, 20 fish (23.65 g±1.51; mean±s.e.m.) were randomly selected and implanted with an intraperitoneal HPT8 MiniChip PIT tag (Biomark, Boise, ID) to track individuals throughout the experiment. Fish were allowed 1 week to recover from tagging to return to homeostasis. Fish were housed at 26°C and 35 ppt in a 300-l tank in a recirculation system outfitted with a biofilter. Fish were fed to satiation daily except for a 48-h fasting period prior to respirometry trials. All experimental protocols and procedures were approved by the University of Texas at Austin Institutional Animal Care and Use Committee (AUP-2017-00200; AUP-2018-00231).

### Intermittent flow respirometry design

Experimental set-up for respirometry was designed as per Svendsen et al. (2016). Eight respirometry chambers (Loligo Systems; Viborg, Denmark) and tubing were measured for volumes (694 ml) and matched to fish mass to achieve a fish-to-water-volume ratio of 1:34±2 (mean±s.e.m.), which is within the best practices for respirometry (Clark et al., 2013; Svendsen et al., 2016). These chambers were set in two equal-sized aerated water baths that overflowed into a common sump. The sump was constantly aerated via an air stone (except during nitrogen bubbling), and held at a constant temperature of 26°C using a titanium heater connected to a Willhi (WH1436A; Bao'an, China) temperature regulator. Water returned to the baths via an Eheim pump (Universal 3400, Deizisau, Germany).

Chambers were outfitted with an incurrent pump (Eheim Compact, Deizisau, Germany) that, when activated during a flush cycle, would replenish the chamber with fresh bath water and wash waste water out via an excurrent tube. A second pump (Mini Brushless 12-volt DC Pump, model #DC20A-1230) acted as a recirculation circuit that ensured mixing within the chamber, and moved water across a submerged fiber-optic oxygen probe (Loligo Systems; Viborg, Denmark). The recirculation pump was always activated. Water flow from both pumps was tempered to allow fish to settle to SMR without active swimming. Oxygen probes were connected to a Witrox-4 (Loligo Systems, Viborg, Denmark) or OXY-4 mini four-channel fiber-optic oxygen transmitter (PreSens, Regensburg, Germany). These machines transmitted information to an automated data acquisition DAQ-M (Loligo Systems, Viborg, Denmark). The DAQ-M connected to the computer program AutoResp (v 2.0, Loligo Systems, Viborg, Denmark) which calculated oxygen consumption (*M*O_2_) using fish mass, water volume and respiratory chamber volume.

To account for bacterial respiration an empty chamber was measured for 60 min before fish introduction and 30 min at the completion of P_crit_ trials. During this time, the cycles were set at 180 s flush, 120 s wait and 2500 s measure. This measurement cycle was longer in order to produce a significant decline in DO for a more precise measurement of background respiration (Svendsen et al., 2016). This total measurement was averaged and a linear growth was assumed. This estimation allowed subtraction of bacterial respiration from fish *M*O_2_ at each time point. In all trials bacterial respiration was low and did not exceed the ‘significant’ threshold of 20% of fish *M*O_2_, as defined by Svendsen et al. (2016).

Prior to introduction into the chamber, fish were fasted for 48 h to ensure they were in a post-prandial state (Clark et al., 2013; Rogers et al., 2016; Regan and Richards, 2017). Fish were weighed and mass was entered into AutoResp to calculate *M*O_2_ (mgO_2_ h^−1^ kg^−1^). Red drum prefer habitats with shelter, so chambers were covered with black plastic to provide a covering for the fish to settle to a calm state. Observations confirmed that the fish spent minimal time swimming. Fish were allowed to acclimate to the chamber for 1 h and *M*O_2_ was measured for at least 24 h for SMR estimation (Clark et al., 2013; Chabot et al., 2016). During this period the cycles were 180 s flush, 120 s wait and 120 s measure. This allowed the fish to deplete oxygen to ∼85%, and the flush cycle to replenish O_2_ to above 95% in the chamber (Svendsen et al., 2016). Measurements of *M*O_2_ were accepted when the linear decrease during the wait and measurement cycles had a value of *r*^2^>0.95. Using R script provided by Chabot et al. (2016), we were able to simultaneously estimate SMR in various commonly used methods in the literature. This analysis included: the mean of the lowest normal distribution; 10th, 15th and 20th percentile values; the average of the lowest 10 measurements; and the mean of the lowest 10% of measurements minus the five lowest measurements. RMR was calculated by averaging *M*O_2_ measurements obtained at >90% O_2_ saturation (18.7 kPa), as per Regan and Richards (2017) to facilitate comparison with current literature.

### Series 1: reduction of pO_2_ in closed circuit respirometry

Initial P_crits_ were determined for all 20 fish using closed circuit respirometry. Following SMR trials, fish were left undisturbed and the incurrent/excurrent water flow was shut off, leaving the recirculation pump to mix water. During these trials, *M*O_2_ was measured every 120 s without a flush or wait cycle. Trials were ended by opening the recirculation pump cycle to the bath to replenish oxygenated water when AutoResp detected a fish had reached 5% (1.0 kPa), which has previously been used in our lab to avoid LOE for most red drum (Ern et al., 2016; Pan et al., 2016), or at the onset of LOE in some cases.

Following the determination of initial P_crit_, the 20 fish were randomly divided into two groups and rested at least 1 week until their second trials. Both groups underwent a second SMR trial and second treatment P_crit_ trial. The first group had a second P_crit_ trial using closed respirometry (*n*=11, mass 21±2 g; mean±s.e.m.). The second group had P_crit_ trials using nitrogen bubbling and intermittent flow respirometry (*n*=9, mass 26±2 g; mean±s.e.m.).

### Reduction of pO_2_ in intermittent flow respirometry

An Oxy-Reg (Loligo Systems, Viborg, Denmark) was set up to control the bubbling of N_2_ into the sump and water bath via a solenoid, air stone, air bubbler and oxygen probe. The Oxy-Reg machine was calibrated before each P_crit_ trial. When the probe detected O_2_ levels at a determined threshold the solenoid would automatically open the valve to gently bubble N_2_ into the water bath and sump and turned off the air bubbler. Measurement cycles were the same as during SMR trials. To prevent surface mixing of O_2_, the water bath and sump were covered with a layer of plastic. The sump pump was turned off at DO levels below 40% to allow N_2_ to displace O_2_ more effectively. An extra pump (Eheim Universal 300) maintained circulation in the bath for adequate water mixing in the baths. This extra set-up was put in place prior to SMR trials, and SMR was run with this set-up dormant until P_crit_ trials so that fish could be left undisturbed and immediately enter P_crit_ trials.

*M*O_2_ was measured in triplicate and averaged at O_2_ saturation levels of 100%, 75%, 50%, 40%, 30%, 25%, 20%, 15%, 10% and 5%, or until LOE. O_2_ saturation was confirmed using an extra AutoResp oxygen probe added to the water. Following trials, fish were transferred to fully oxygenated water for recovery. DO (%) was converted to O_2_ partial pressure (pO_2_) in kPa using a conversion factor of 2.051×10^−1^ kPa %^−1^.

### P_crit_ determination

P_crit_ was calculated from respirometry datasets using several common methodologies (see Table 1). The primary method involved identifying the point of intersection between the O_2_ conformation phase of the trace and SMR, as previously described (McBryan et al., 2016; Snyder et al., 2016). In all cases, the linear regression of the O_2_ conformation phase exhibited an *r*^2^≥0.95. A second method used the same approach with the exception that the pre-determined RMR (averaged *M*O_2_ when pO_2_>18.7 kPa over 24-h prior to P_crit_ trials) value was used to the define the point of intersection. Alternative approaches included the two segmented straight line (BSR) and mid-point approaches of Yeager and Ultsch (1989) and the non-linear broken line (segmented) method of Muggeo (2003). Briefly, BSR calculates P_crit_ by fitting two linear regressions to the data of a P_crit_ trace, as opposed to the 24-h SMR estimate (see Fig. 4). Segmented is similar in that two lines are fit to a P_crit_ trace; however, a point of intersection is not used to anchor the two regression lines, leaving a potential ‘gap’ where the two lines meet. P_crit_ is estimated as the point with the smallest gap between the two segmented lines (Muggeo 2003). Note that these methods do not use the *M*O_2_ data points collected in the 24-h prior to the onset of P_crit_ trials. All calculations were performed using common R packages rMR (Moulton, 2018) and respR (Harianto et al., 2019).

### Series 2: waste product measurements

Two additional series of SMR and P_crit_ experiments (*N*=16; mass=24 g±1) were conducted for both intermittent and closed respirometry (22 ppt and 26°C) to specifically assess waste build-up. The first series of trials included a complete SMR and P_crit_ trial (ending at 1.0 kPa) as described above, and water samples were collected from the chamber at the conclusion of the trial. The second series allowed the chamber oxygen level to reach 30% air saturation (6.2 kPa) at which point water samples were collected. Note that the second trial was intended to isolate only the aerobic portion of the P_crit_ trial. Water samples from the chamber were collected by disconnecting the recirculating pump and collecting the first 50 ml. Samples were taken at the beginning of SMR trials, the beginning of a P_crit_ trial (end of SMR trials) and the conclusion of trials (either 6.2 or 1.0 kPa). pH was measured immediately using a hand-held Accumet AB15 pH meter (Thermo Fisher Scientific), after which samples were capped and stored at 4°C until determination of ammonia and total alkalinity assays. Assays for total alkalinity and ammonia were performed within 72 h of collection.

Total alkalinity measurements were performed using a Total Alkalinity Titrator System (model AS-ALK2, Apollo SciTech, Newark, DE), and all samples were tested in duplicate and averaged. Total alkalinity was combined with sample pH, salinity and temperature to calculate total CO_2_ (TCO_2_ in µmol l^−1^) and the partial pressure of CO_2_ (pCO_2_ in µatm) using CO2Sys_v2.1 (Pierrot et al., 2006). pCO_2_ was converted from µatm to kPa using a conversion factor of 1.013×10^−4^ kPa µatm^−1^. Total ammonia concentration (µmol l^−1^) was determined using a standard colorimetric assay (Verdouw et al., 1978).

To place calculated pCO_2_ values in an environmental context, a long-term data series that monitors water quality every 15 min was retrieved from the NOAA National Estuarine Research Reserve System (NERRS, 2013) Centralized Data Management Office for May 2012 to September 2012. Water quality data (salinity, DO, pH, etc.) were downloaded from the Apalachicola Bay NERR station in the Northern Gulf of Mexico.

### Statistical analysis

A two-way mixed model ANOVA assuming unequal variance (α=0.05) was used to assess differences between closed and intermittent flow P_crit_ measurements. Changes between the first and second measurements for an individual were performed using RM while differences between experimental treatments (closed and intermittent flow) were unpaired. To see if differences in SMR calculation would affect P_crit_ outcomes, a two-way RM ANOVA assuming unequal variance (α=0.05) was conducted with type of SMR calculation and treatment as factors. Within each type of SMR calculation a Student's *t-*test (two-tailed, α=0.5) was conducted to see the effect of treatment on P_crit_. For waste differences, a two-way ANOVA was performed with time point and treatment as factors. When a significant interaction was found, a Tukey HSD post-hoc test revealed differences between groups.
